# The Making of a Tropical Disease: A Short History of Malaria

**DOI:** 10.3201/eid1410.080834

**Published:** 2008-10

**Affiliations:** Centers for Disease Control and Prevention, Atlanta, Georgia, USA (retired)

This publication is one of a series published by the Johns Hopkins University Press on biographies of disease. Earlier volumes were Mania: A Short History of Bipolar Disorder and Dropsy, Dialysis, and Transplant: A Short History of Failing Kidneys. Malaria is clearly a worthy subject in this ambitious series.

The preface sets the stage for the treatise on malaria and establishes the author’s interest and qualifications for writing the book. The first chapter provides a reasonable scenario for establishing Africa as the place of origin of human malaria parasites and the probable movement of the organisms with movements of early humans from Africa through southern Asia and eventually to the Pacific Islands.

The reviewer was somewhat uncomfortable with the complete absence of any discussion of the evolution of *Plasmodium* species in nonhuman primates because those parasites are clearly closely related to those found in humans. The statement that there are 4 species of malaria parasites that infect humans is inaccurate. Recent reports of the extensive occurrence of natural human infections with *P*. *knowlesi* in Borneo and the Philippines are an issue that warrants attention. (Experimental infections in humans with malaria parasites from nonhuman primates in Asia do not need to be detailed here.) Fortunately, some weaknesses in the discussion of the evolution of primate malaria parasites do not seriously detract from the detailed and well-written story of malaria as a human disease.

The movement of malaria into northern areas and its eventual retreat back to the tropics is well told and clearly addresses the central theme of “the making of a tropical disease.” The discussions of the long history of malaria control efforts directed toward the vector and, to a lesser extent, the parasite without what the author considers adequate attention to the social aspects of malaria occurrence are well structured. The recounting of the disastrous Global Malaria Eradication effort is must reading for anyone interested in human malaria. The reviewer experienced this effort personally but continues to be fascinated with this extraordinary story.

The discussion of the current program, “Roll Back Malaria,” is an essential part of this story. This ongoing and massive effort to bring malaria under control is multifaceted and heavily funded, and its eventual outcome may well inspire Dr. Packard to write an addendum to this interesting book.

The author’s focus on poverty and its contribution to the continued presence of malaria in endemic areas, especially Africa, is well presented. There is no doubt that war, famine, political upheaval, and human poverty are primary issues in the continued presence of malaria as a major cause of illness and death. Unfortunately, this book does not offer a solution to these issues. This book should be read by and on the shelf of anyone working in or generally interested in the place of malaria in human history.

